# Using Restriction Endonuclease, Protection, Selection, and Amplification to Identify Preferred DNA-Binding Sequences of Microbial Transcription Factors

**DOI:** 10.1128/spectrum.04397-22

**Published:** 2023-01-05

**Authors:** John K. Barrows, Michael W. Van Dyke

**Affiliations:** a Department of Chemistry and Biochemistry, Kennesaw State University, Kennesaw, Georgia, USA; Forschungszentrum Jülich GmbH

**Keywords:** iterative selection methods, protein-DNA interactions, *Thermus thermophilus*, transcriptional regulation

## Abstract

Regulation of gene expression is a vital component of cellular biology. Transcription factor proteins often bind regulatory DNA sequences upstream of transcription start sites to facilitate the activation or repression of RNA polymerase. Research laboratories have devoted many projects to understanding the transcription regulatory networks for transcription factors, as these regulated genes provide critical insight into the biology of the host organism. Various *in vivo* and *in vitro* assays have been developed to elucidate transcription regulatory networks. Several assays, including SELEX-seq and ChIP-seq, capture DNA-bound transcription factors to determine the preferred DNA-binding sequences, which can then be mapped to the host organism’s genome to identify candidate regulatory genes. In this protocol, we describe an alternative *in vitro*, iterative selection approach to ascertaining DNA-binding sequences of a transcription factor of interest using restriction endonuclease, protection, selection, and amplification (REPSA). Contrary to traditional antibody-based capture methods, REPSA selects for transcription factor-bound DNA sequences by challenging binding reactions with a type IIS restriction endonuclease. Cleavage-resistant DNA species are amplified by PCR and then used as inputs for the next round of REPSA. This process is repeated until a protected DNA species is observed by gel electrophoresis, which is an indication of a successful REPSA experiment. Subsequent high-throughput sequencing of REPSA-selected DNAs accompanied by motif discovery and scanning analyses can be used for determining transcription factor consensus binding sequences and potential regulated genes, providing critical first steps in determining organisms’ transcription regulatory networks.

**IMPORTANCE** Transcription regulatory proteins are an essential class of proteins that help maintain cellular homeostasis by adapting the transcriptome based on environmental cues. Dysregulation of transcription factors can lead to diseases such as cancer, and many eukaryotic and prokaryotic transcription factors have become enticing therapeutic targets. Additionally, in many understudied organisms, the transcription regulatory networks for uncharacterized transcription factors remain unknown. As such, the need for experimental techniques to establish transcription regulatory networks is paramount. Here, we describe a step-by-step protocol for REPSA, an inexpensive, iterative selection technique to identify transcription factor-binding sequences without the need for antibody-based capture methods.

## INTRODUCTION

Regulation of gene expression is a fundamental biological mechanism for both prokaryotic and eukaryotic organisms. Cells have evolved various means to sense extracellular and intracellular stimuli and adapt their transcriptome accordingly. Transcription regulatory proteins, often referred to as transcription factors, are an essential class of proteins that help control gene expression, predominantly through modulating RNA polymerase activity. Extensive research has been conducted to identify transcription regulatory networks for transcription factors, which provide information on the transcription factor’s biological function (1). Often, studies involving transcription regulatory network identification begin with differential gene expression analysis between a reference and transcription factor mutant strain using high-throughput means such as RNA sequencing or DNA microarrays. These transcriptome-wide analyses yield hundreds of dysregulated genes, when only a small fraction are directly regulated by the transcription factor in question (2). Many transcription factors, especially from prokaryotic organisms, recognize specific DNA motifs often found in the promoters of genes within their transcription regulatory network (3). As such, determination of transcription regulatory networks should, at minimum, include evidence of sequence-specific DNA binding and dysregulated gene expression *in vivo* in transcription factor knockout or overexpression strains.

Identifying the preferred DNA-binding sequence of a transcription factor presents an alternative starting strategy to discovering transcription regulatory networks, compared to transcriptome-wide analyses. Indeed, several high-throughput experimental techniques have been developed to identify DNA-binding sequences of transcription factors *in vivo* and *in vitro*. *In vivo*-based methods include chromatin immunoprecipitation (ChIP)-seq (4) and ChIP-chip (5), wherein a transcription factor of interest is immunoprecipitated from cross-linked biological samples and the resulting protein-bound DNA sequences are analyzed by high-throughput sequencing (ChIP-seq) or DNA microarray analysis (ChIP-chip). Common *in vitro* methodologies for identifying the preferred DNA-binding sequences of transcription factors include systematic evolution of ligands by exponential enrichment (SELEX) accompanied with high-throughput sequencing (SELEX-seq) and high-density double-stranded DNA (dsDNA) microarrays (6). For SELEX-seq, affinity-tagged transcription factors are incubated with DNA templates containing a region of randomized sequences. Protein-bound DNAs are separated from unbound DNAs through affinity purification and the resulting DNAs are amplified by PCR. This process is repeated until most DNAs remaining after PCR associate with the tested transcription factor; the resulting DNAs are then analyzed through high-throughput sequencing technologies (7, 8). Separation of protein-bound DNAs in SELEX-seq has also been achieved through electromobility shift assays (EMSAs) (9) and filter-binding assays (10). Another *in vitro* approach, high-density dsDNA microarrays, can be used to screen large libraries of DNA sequences with a recombinant transcription factor (11). In this method, protein-bound DNA sequences are identified though fluorescently conjugated antibodies that recognize the transcription factor of interest. Ultimately, consensus DNA-binding motifs identified by these techniques can be mapped to the host organism’s genome, providing a comprehensive list of potential DNA-binding locations. The location of these sequences relative to transcription start sites can give an idea of which gene(s) the transcription factor may regulate, which can be used for experimental validation.

**FIG 1:**
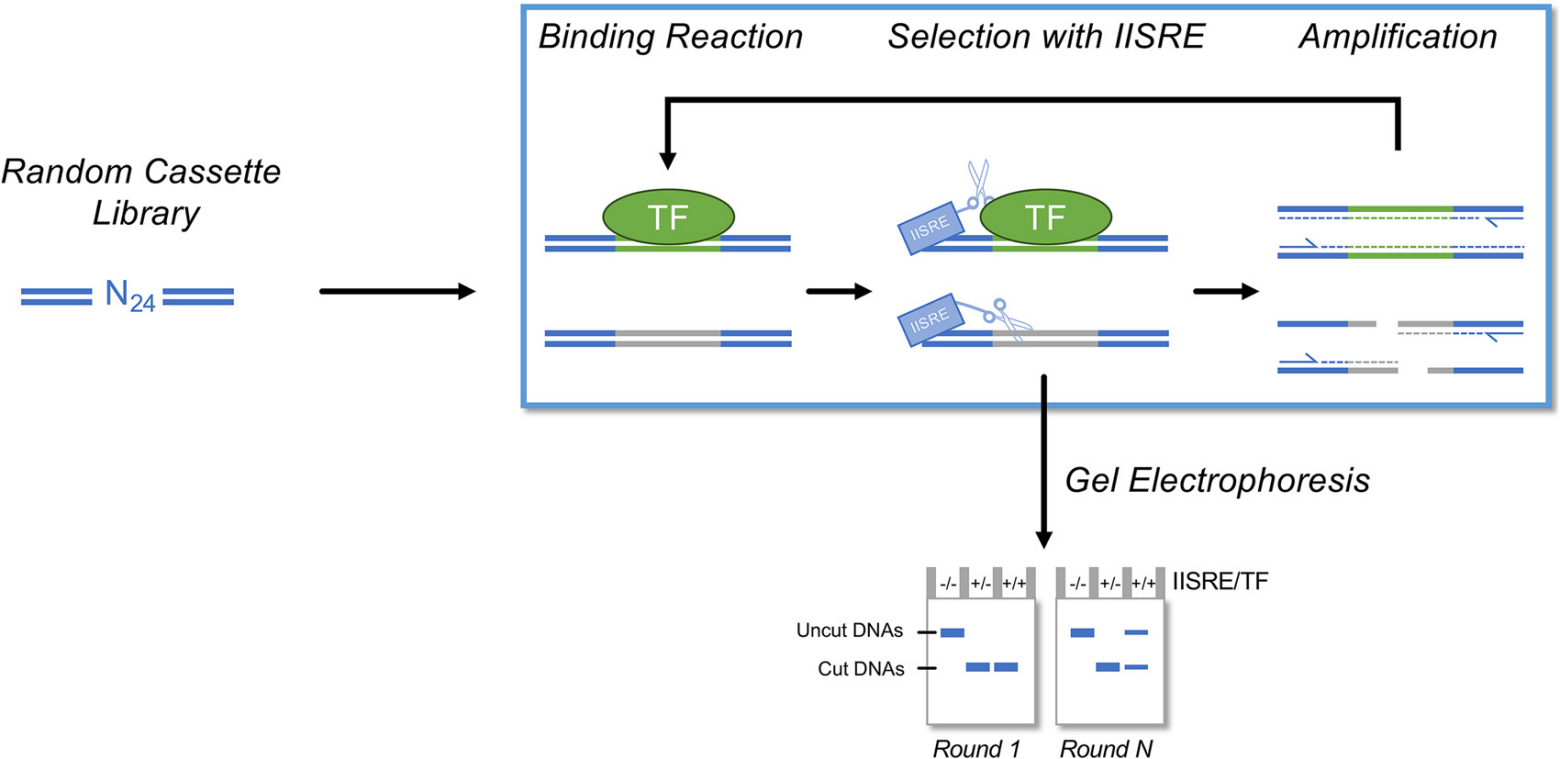
REPSA overview. PCR-generated selection libraries containing defined flanking sequences and an internal cassette of random nucleotides are incubated with a transcription factor of interest and then challenged with a type IIS restriction endonuclease (IISRE). Cleavage-protected DNA species are amplified by PCR, purified, and then used as inputs for the next round of REPSA (blue box). After each round, samples from reactions containing no transcription factor and no IISRE (−/−), no transcription factor with IISRE (+/−), and with both transcription factor and IISRE (+/+) are analyzed by gel electrophoresis (bottom). Rounds of REPSA are continued until a protected DNA species is observed in the +/+ sample.

In this article, we provide an updated protocol to an alternative, *in vitro* iterative selection assay, restriction endonuclease, protection, selection, and amplification (REPSA; Fig. 1) (12). Like SELEX, REPSA assays transcription factor binding to a library of DNAs containing a region of random nucleotides. However, unlike the affinity-purification step used in SELEX, REPSA selects for protein-bound DNA sequences by challenging transcription factor-DNA reactions with a type IIS restriction endonuclease (IISRE). IISREs differ from their more common type II counterparts by cleaving dsDNA a fixed distance away from their binding site (13). Therefore, IISREs will cleave dsDNA without regard for sequence specificity at the cleavage site. DNA templates used for selection in REPSA contain IISRE recognition sequences flanking a region of randomized nucleotides, so that the IISRE digestion occurs within the randomized region. DNAs containing bound transcription factors within the randomized region will prevent restriction endonuclease cleavage, resulting in a protected, full-length DNA template. After reactions are challenged with an IISRE, the resulting DNAs are amplified by PCR. Cleaved DNA species will be unable to produce a complete amplicon, while protected DNA species will be amplified. This process is repeated with the purified PCR products until a protected DNA species is observed by gel electrophoresis. These protected DNAs may then be sequenced and analyzed for recurrent motifs, thereby yielding transcription factor consensus binding sequences. Although this protocol describes the use of REPSA for transcription factor DNA-binding specifically, REPSA can be used with a variety of other ligands, including small molecules and nucleic acids (14, 15).

## RESULTS AND DISCUSSION

In the example protocol presented in the following Materials and Methods section, we describe the use of REPSA to identify preferred DNA-binding sequences for a transcription factor of interest. REPSA provides an alternative approach to traditional antibody-based methods, such as ChIP-seq. For many understudied transcription factors, commercial antibodies do not exist, and thus, REPSA presents an enticing procedure to analyze DNA-binding sequences for these proteins. REPSA also does not require affinity-tagged recombinant proteins or separation of DNA-protein complexes by electrophoresis, as with traditional SELEX experiments. Notably, REPSA is not exclusive to transcription factor ligands and has been experimentally validated to find DNA-binding sequences for non-transcription factor proteins (16), nucleic acids (14), and small molecules (17, 18).

Each round of REPSA contains two control reactions that lack the transcription factor of interest. One of these control reactions is treated with an IISRE (cut sample), while the other is untreated (uncut sample). The purpose of these reactions is to show the relative migration of uncut and cut DNA species by electrophoresis, as well as to directly determine the efficiency of DNA cleavage by the IISRE. In the first round of REPSA, one should observe near-100% digestion efficiency for the cut control sample and +TF sample. If one observes substantial cleavage protection in the +TF sample, then this is likely due to nonspecific binding of the transcription factor to DNA or specific binding to a sequence within the flanking regions of one’s selection template. Nonspecific DNA binding can be common for proteins with high isoelectric points (>8), which may have a strong, nonspecific affinity to the negatively charged, phosphate backbone of DNA. To remedy this, one should decrease the concentration of transcription factor or add unlabeled DNA (such as lambda-DNA or poly[dI-dC]) until the amount of cleavage-resistant species in round 1 is <10% of the total DNA population. If one observes substantial cleavage protection in round 1 of REPSA in the cut sample, then the concentration of IISRE should be increased or the amount of input DNA should be reduced. It is worth noting that the mechanism of action for FokI cleavage requires two FokI monomers to interact at the cleavage site, and this preferentially occurs between DNA-bound monomers (19). Thus, the efficiency of FokI cleavage can be directly related to selection template concentration. Although the cleavage efficiency of FokI presented in this protocol’s setup is high, if one desires to substantially decrease the template concentration for REPSA, it may significantly reduce FokI cleavage. One possible remedy to enhance FokI cleavage in this scenario may be to supplement binding reactions with unlabeled double-stranded oligonucleotides containing the FokI recognition sequence.

Most transcription factor-binding sequences are between 5 and 15 bp long (20). We typically add ~3 ng of our selection template to round 1 reactions, which approximates to ~4 × 10^10^ DNA molecules. This gives a reasonable representation of all possible combinations of 15 bp, which is ~5 × 10^8^ DNA sequences. However, binding sequences of >17 bp may not be adequately represented with our current protocol. Exploring these would necessitate commensurately increased DNA library amounts and reaction volumes.

**FIG 2:**
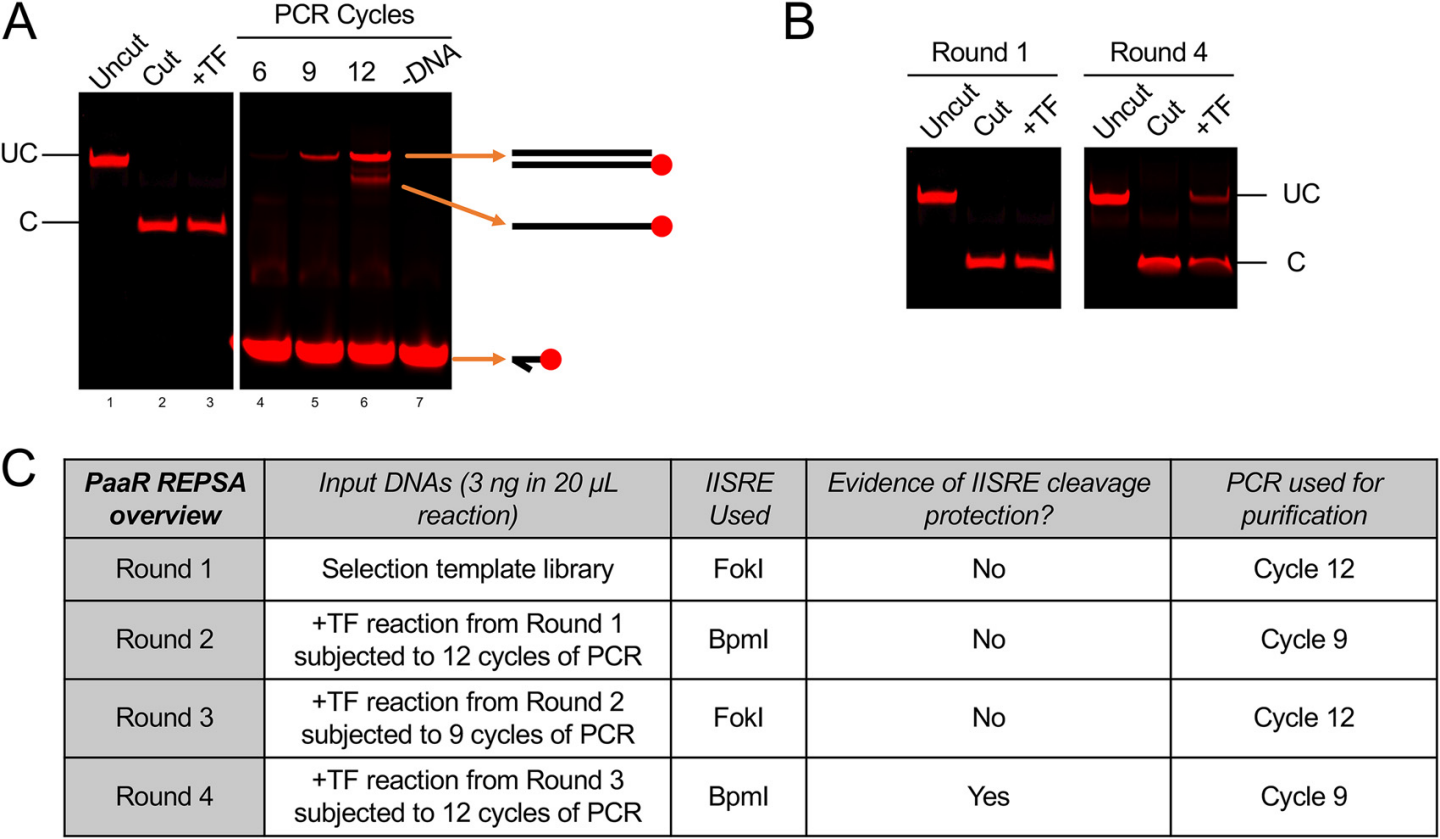
REPSA with Thermus thermophilus PaaR. (A) Binding/IISRE reaction (lanes 1 to 3) and PCR (lanes 4 to 7) samples from round 1 of REPSA were separated by native PAGE and visualized using a LI-COR Odyssey Imager. Uncut (UC) and cut (C) DNA bands are denoted for the binding/IISRE reaction samples. Different DNA species from the PCR samples are labeled. The intensities between the binding/IISRE reaction and PCR samples are modified for clarity (denoted by the gap between lanes 3 and 4). An equal intensity gel containing all 7 samples is presented in Fig. S1A in the supplemental material. (Uncut) Reactions lacked PaaR and IISRE. (Cut) Reactions lacked PaaR and were challenged with 0.4 units IISRE. (+TF) Reactions contained 100 nM PaaR and were challenged with 0.4 units IISRE. (B) Binding/IISRE reaction samples from rounds 1 and 4 of REPSA are presented. Uncut and cut DNA bands are denoted. Note the presence of a transcription factor (TF)-dependent, protected DNA species in round 4. (C) Overview of each round of REPSA used for the sample experiment.

**FIG 3:**
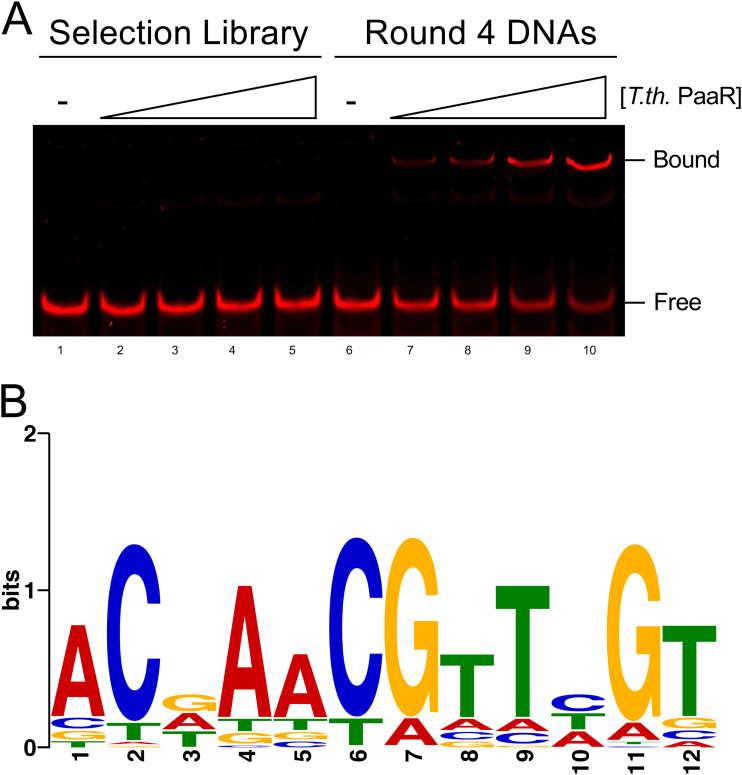
Round 4 DNAs specifically associate with PaaR. (A) DNAs from the selection library used for round 1 of REPSA or DNAs amplified after round 4 of REPSA were incubated with 0, 37.5, 75, 150, or 300 nM monomeric Thermus thermophilus PaaR. DNA complexes were separated by native PAGE and visualized using a LI-COR Odyssey Imager. Free and PaaR-bound DNA complexes are indicated. (B) Round 4 DNAs were given sequence barcodes by fusion PCR to allow for massively parallel semiconductor sequencing using an Ion Torrent Personal Genome Machine. The resulting sequences were trimmed to yield only the 24-bp variable region. Approximately 3,000 sequences were then input into the Web version of Multiple Em for Motif Elicitation (MEME) v5.5.0 using a palindrome filter. The sequence logo of the most significant motif (E value, 5.0 × 10^−1225^) is shown.

Identification of a protected DNA population in later rounds of REPSA during gel electrophoresis is an indication of a successful REPSA experiment. Normally, protected DNA species only account for 30% to 60% of the entire DNA population (see Fig. 2B). However, after the subsequent PCR, we see that ~70% of the round 4 DNA sequences can associate with our transcription factor by EMSA (Fig. 3A), showing that most of our final REPSA-identified DNA population contains motifs suitable to transcription factor binding. Identification of consensus DNA-binding motifs requires high-throughput sequencing of the DNA products produced from a cleavage-resistant round of REPSA. This can be achieved by amplifying DNA pools with primers suitable for Illumina-based sequencing technology (described in more detail on the Illumina website) or outsourcing to a DNA-sequencing company. Significant DNA motifs (as shown in Fig. 3B), as well as potential genomic binding sequences, can be elucidated from the high-throughput sequencing results using motif discovery analysis tools presented in the MEME suite (21). Examples of applications of these approaches to identify potential transcription factor-regulated genes may be found in our prior studies (22–26). Potential transcription factor-binding sequences should be experimentally validated, ideally in both *in vitro* and *in vivo* settings.

Collectively, REPSA is an established, cost-effective technique to identify preferred DNA-binding sequences using nanomolar concentrations of protein. Understanding the DNA selectivity of transcription factors can help elucidate transcription regulatory networks and provide valuable insight into their biological function.

## MATERIALS AND METHODS

### Materials and equipment.

**FIG 4:**
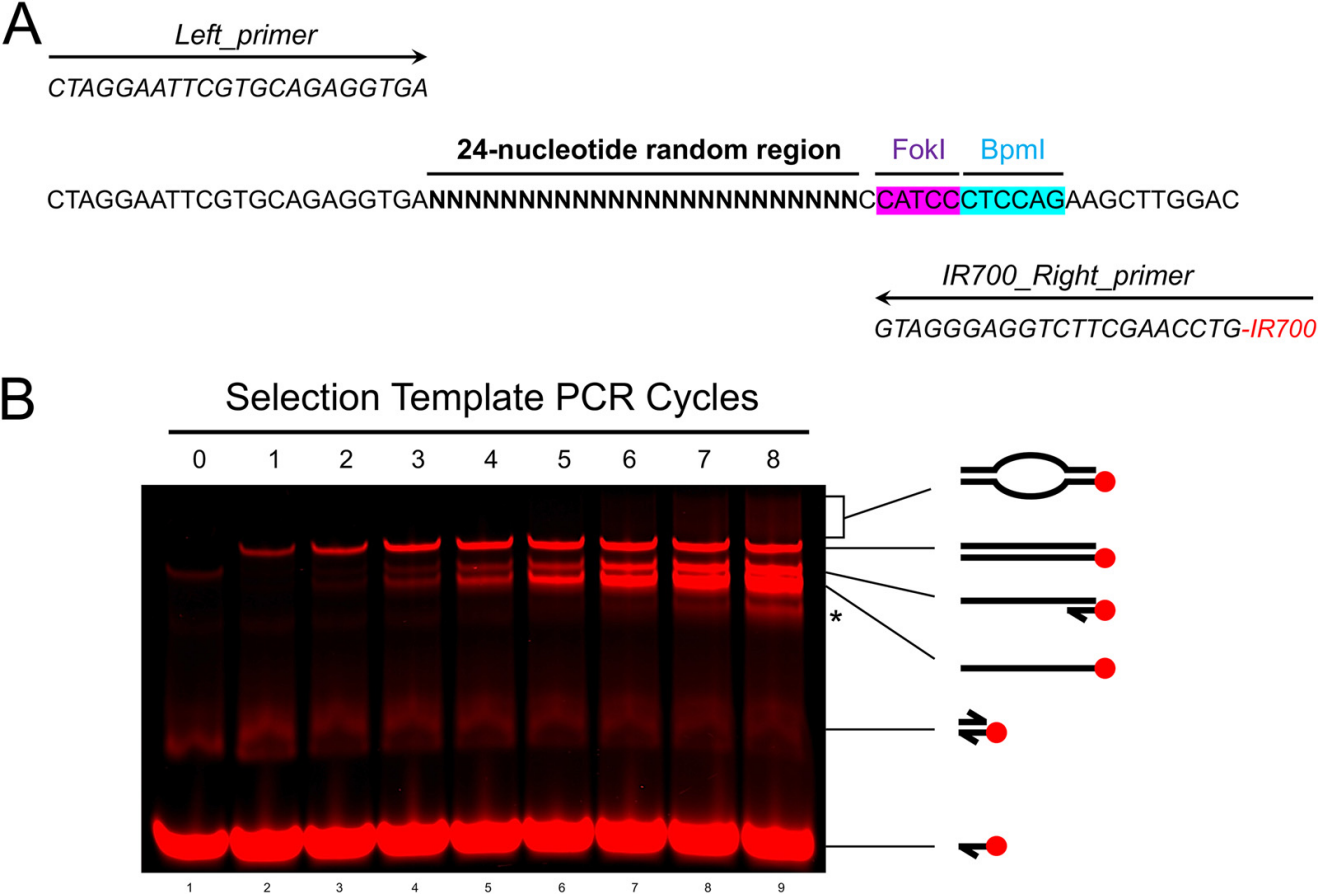
DNA selection template design and PCR amplification. (A) Schematic of selection template oligonucleotide used throughout this study, as well as the primers used for PCR amplification. The selection template oligonucleotide and primers were purchased from Integrated DNA Technologies. “N” represents random nucleotides (25% A/T/G/C). The selection template oligonucleotide was purified under standard desalting conditions. The right primer was conjugated with 5′ IRDye 700 to allow visualization using near-infrared fluorescent imaging systems. (B) The DNAs described in panel A were subjected to up to 8 cycles of PCR. Samples were withdrawn after each cycle, resolved by native PAGE, and visualized using a LI-COR Odyssey Imager. Representative images depicting the DNA species of visible bands are shown. The asterisk (*) indicates an ssDNA product, likely resulting from annealing within the random cassette region.

- Selection template oligonucleotides and primers used for fluorescently labeled PCR amplification (examples are presented in Fig. 4A).
- PCR reagents: Our laboratory typically uses *Taq* polymerase from New England Biolabs (NEB) with standard *Taq* buffer (NEB).
- DNA clean and concentrator kit v5 (Zymo Research).
- Type IIS restriction enzymes (at least two) with accompanying reaction buffers. We typically use FokI and BpmI (NEB) in reactions containing 1× CutSmart buffer (NEB).
- Purified protein of interest (affinity tags are not required for REPSA but may be necessary for protein purification).
- 6× gel loading dye, orange (NEB). Denoted as “orange loading dye” throughout the article.

Machines needed (machines used for the example presented in this article are shown in parentheses):

- Thermocycler (Bio-Rad C1000 Touch).
- Qubit fluorometer (Invitrogen Qubit 3).
- Gel electrophoresis system (Bio-Rad Mini-Protean Tetra cell).
- Near-infrared fluorescent imaging system (LI-COR Odyssey).

### Design of selection template.

REPSA consists of several “rounds” in which a set of DNAs is incubated with a transcription factor of interest and then challenged with an IISRE. The first round of REPSA utilizes a selection library of dsDNA templates containing a region of random nucleotide sequences. Each round selects for preferred DNA-binding sequences, which are then subjected to PCR and used as inputs for the subsequent round of REPSA. To create an initial selection library, selection template oligonucleotides must first be developed. These oligonucleotides should contain a region of randomized nucleotides flanked by defined DNA sequences. Our laboratory has had success with randomized sequences ranging from 14 to 26 nucleotides long. Flanking regions should contain IISRE recognition sequences and be long enough to design primers for PCR amplification of the selection template. It is important to note that primers designed for template amplification should contain the IISRE recognition sequences. Having these sites within the primers avoids unwanted mutagenesis of the IISRE recognition sequences that would prevent DNA cleavage. To visualize PCR products, it is advantageous to design one unlabeled primer and one primer that is conjugated to a fluorescent dye at its 5′ end, allowing detection by near-infrared fluorescent imaging systems (27). Such conjugates provide greater sensitivity than most indirect staining methods and avoid the hazards of mutagenic dyes or radioactivity. Selection templates should contain at least two IISRE recognition sequences, as our laboratory has observed IISRE-specific cleavage-resistant DNA species that can be selected for by using the same IISRE each round (22). Templates and primers can be easily designed and purchased through a desired custom DNA oligonucleotide manufacturer (ours were obtained from Integrated DNA Technologies). A sample selection template oligonucleotide and accompanying primers for PCR amplification that will be used throughout this protocol are presented in Fig. 4A.

### Preparation of the REPSA template.

Selection templates must undergo PCR to create correctly annealed duplex DNAs. Annealing of complementary selection templates in the absence of PCR would result in duplexes that contain many mismatched base pairs in the random region. However, due to defined flanking regions yet highly variable, random central sequences, selection templates should not undergo extended cycles of PCR. Doing so will result in a high population of single-stranded DNAs (ssDNAs) and incorrectly annealed heteroduplex or “bubble” species (28). To identify the optimal PCR cycles, we performed a PCR with a selection template oligonucleotide and analyzed the DNA products after each cycle, as described below.

1. Set up a PCR. This protocol uses New England Biolabs *Taq* polymerase with standard *Taq* buffer. The final 50-μL PCRs should contain 1× standard *Taq* buffer, 200 μM dNTPs (deoxynucleoside triphosphates), 0.2 μM 5′ IRDye 700-labeled primer, 0.24 μM unlabeled primer, 4 ng template oligonucleotide, and 0.025 units/μL *Taq* polymerase. The slight increase in unlabeled primer helps reduce labeled ssDNA species during DNA analysis.
2. Remove 1 μL from the PCR and mix with 5 μL 1.2× orange loading buffer for a “0 cycle” sample.
3. Use a thermocycler to perform the PCR protocol presented above (Table 1). Annealing temperatures should be determined based on the properties of the primers used.
4. Remove 1 μL from the PCR and mix with 5 μL 1.2× orange loading buffer after each cycle (during the 4°C hold step).
5. To analyze DNA products, separate the samples using 10% native PAGE and visualize the DNA using a near-infrared fluorescent imaging system. A sample workflow for identifying the optimal number of PCR cycles for selection template oligonucleotides is presented in Fig. 4B. The optimal number of PCR cycles should yield the most dsDNA product, while producing minimal ssDNA and bubble species. For the example in Fig. 4B, the optimal number of PCR cycles would be 3, since this produces similar quantities of dsDNA product compared to later cycles but contains only a small fraction of ssDNAs.
6. Set up a PCR as indicated in step 1, but now run the PCR protocol for the ideal number of cycles determined experimentally in step 5 (remove the 4°C hold steps).
7. Purify the PCR products. To do so, our laboratory utilizes DNA clean and concentrator kits from Zymo Research, following the manufacturer’s protocol. DNA purification in this manner results in a substantial removal of unincorporated primer species that may hinder REPSA selection (see Fig. S1 in the supplemental material; compare lanes 1 to 3 to lanes 4 to 7).
8. Purified DNA products can then be quantified using a Qubit fluorometer (29). Typical yields from a 50-μL, 3- to 5-cycle PCR is approximately 20 to 40 ng dsDNA. Multiple PCRs can be purified together if a higher yield is desired. This purified dsDNA selection library is now ready for round 1 of REPSA.

**TABLE 1:** Thermocycler settings for selection template PCR

Step	Description	Temp (°C)	Time
1	Initial denaturation	95	2 min
2	Denaturation	95	30 s
3	Annealing	55	30 s
4	Extension	68	60 s
5	Sample isolation	4	Hold
6	Repeat steps 2–5; ×7		

### Round 1 of REPSA.

**(i) Binding reactions.** Binding reactions allow for the transcription factor of interest to interact with the inputted DNA sequences.

1. Set up binding reactions in 0.2-mL PCR tubes. Typical 20-μL volume reactions contain the following: 1× CutSmart buffer (NEB), 3 ng selection template DNAs, and 20 to 100 nM of the transcription factor of interest. This tube should be labeled “+TF,” as it contains the tested transcription factor. Any dilutions of the transcription factor of interest from its stock are performed with 1× CutSmart buffer.

Note: The addition of a reducing agent, such as 1 mM dithiothreitol, may be necessary in binding reactions for transcription factors containing cysteine residues.

2. Create two control reactions (labeled “uncut” and “cut”) that are identical to the binding reaction described in step 1 but lack the transcription factor. These will be used to determine the cleavage efficiency of the IISRE and to act as DNA markers for uncut and cut dsDNA templates.
3. Incubate all reactions at a physiological temperature that promotes binding for 20 min.

In the examples used throughout this protocol, REPSA is performed using a thermophilic, TetR family transcription regulator, PaaR, from Thermus thermophilus HB8 (23, 30). As such, binding reactions are conducted at 55°C. Binding temperatures for mesothermic proteins should mimic their host organisms’ preferred growth temperature. For example, binding reactions for E. coli proteins should occur at 37°C. Note: Binding reactions at temperatures higher than 55°C may result in partial denaturation of the selection templates and should be avoided, as incompletely annealed DNA in the randomized region will not be cut by IISREs.

**(ii) Restriction endonuclease addition.** Type IIS restriction endonucleases (IISREs) are used in REPSA to digest unbound dsDNA templates. If a protein of interest binds with high affinity to a sequence within the random nucleotide region, then the protein-DNA complex will create a barrier to DNA cleavage by the IISRE. In our laboratory, the most common IISREs used in REPSA are FokI and BpmI, both of which are most active at 37°C. Note: IISRE enzymes should be alternated in sequential rounds of REPSA to avoid selection of IISRE-specific cleavage-resistant DNAs.

4. Transfer control and binding reaction tubes to 37°C for 5 min. This step may be skipped if the binding reaction occurred at 37°C.
5. Add 0.4 units of IISRE to both the binding reaction (+TF) and one control reaction (cut). We suggest pipetting up and down using a P20 pipette after IISRE addition to ensure thorough mixing.

Typically, we dilute IISRE stocks with 1× CutSmart buffer to a final concentration of 0.4 units/μL and then add 1 μL of the dilution to the respective reactions.

6. Incubate all reactions at 37°C for 5 min.
7. Transfer the reactions to ice. This will stop IISRE digestion.
8. Remove 3 μL of each reaction (uncut, cut, and +TF) and mix with 15 μL 1.2× orange loading dye for DNA analysis (see “DNA analysis and purification”).

**(iii) PCR amplification of binding reaction samples.** Samples from the +TF binding reaction will be subject to PCR amplification. Cleaved DNA templates will fail to produce a complete amplicon, while intact DNAs should create full-length DNA products (Fig. 1). IISRE digestion is not 100% efficient; therefore, after the first round of REPSA, most remaining intact DNA templates are likely to be DNAs from the selection library that evaded IISRE digestion. DNAs protected from IISRE cleavage due to transcription factor binding are only a small fraction of these uncleaved DNAs from round 1. Later rounds of REPSA will increase the selection of transcription factor-bound DNA sequences. As with the selection oligonucleotide PCR, the binding reaction PCR will be assayed at multiple (typically, 6, 9, and 12) PCR cycles. This allows the identification of PCR products that will be best suited for the subsequent round of REPSA (i.e., maximal quantity of duplex DNA, minimal presence of single-stranded or bubble DNAs).

9. Create a PCR master mix solution for 4.5× reaction volumes containing the components listed in Table 2.
10. Aliquot 23 μL from the master mix into a 0.2-mL PCR tube containing 2 μL water. This tube will be a “−DNA” sample and used to identify any DNA contaminates in the PCR solution setup.
11. Add 7 μL of the ice-cold +TF binding reaction to the remaining PCR master mix and mix thoroughly by pipetting up and down with a P200 pipette. Aliquot 25 μL of this mixture into three 0.2-mL PCR tubes labeled “6 cycles,” “9 cycles,” and “12 cycles”.
12. Perform the PCR protocol listed in Table 3. Hold steps at 4°C are introduced after 6, 9, and 12 cycles. After 6 cycles have completed, remove the PCR tube labeled “6 cycles” from the thermocycler and place it on ice. Do the same for the other tubes after their respective PCR cycles are completed. Let the −DNA reaction proceed for 12 PCR cycles. As mentioned previously, annealing temperatures should be determined based on the melting temperatures of the primers used.
13. Once the PCR program is completed, remove 0.5 μL of each PCR and mix it with 5 μL 1.2× orange loading dye for DNA analysis.

**TABLE 2:** PCR master mix for binding reaction amplification

Component	Amt for 25-μL reaction (μL)	
	1×	4.5×
10× standard *Taq* buffer	2.5	11.25
10 mM dNTPs	0.5	2.25
2 μM left_primer^*a*^	3	13.5
2 μM IR700_right_primer^*a*^	2.5	11.25
Water	14.37	66.67
5 units/μL *Taq* DNA polymerase	0.13	0.59
Total	23	103.5

a Left and right primers, see Fig. 4A.

**TABLE 3:** Thermocycler settings for binding reaction PCR

Step	Temp (°C)	Time
1	95	2 min
2	95	30 s
3	55	30 s
4	68	60 s
5	Repeat steps 2–4; ×5	
6	68	60 s
7	4	Hold
8	Repeat steps 2–4; ×3	
9	68	60 s
10	4	Hold
11	Repeat steps 2–4; ×3	
12	68	60 s
13	4	Hold

**(iv) DNA analysis and purification.**

14. Separate the samples by 10% native PAGE and image them using a near-infrared fluorescent imager (31). A sample DNA analysis gel from round 1 of REPSA using Thermus thermophilus PaaR and the selection template presented in Fig. 4A are shown in Fig. 2A. It is important to note that most DNAs in reactions containing the tested transcription factor should be digested in round 1 (Fig. 2A, lane 3; see Results and Discussion).
15. Analyze the DNA products from the 6, 9, and 12 cycles of PCR, as done previously with the selection template PCR (Fig. 4B). Samples containing the most dsDNA template are ideal. If the dsDNA amounts are equivalent between samples, choose the sample with the smallest amount of ssDNA and bubble species. For the example presented in Fig. 2A, the best sample is cycle 12 (lane 6), as this sample has the most dsDNA template with a minor ssDNA contaminate species.

Note: Large quantities of ssDNA or bubble species should be avoided, as they are unable to be digested by IISREs. However, these contaminates are eventually removed following the next round’s PCR step and subsequent IISRE digest.

16. Purify the remaining PCR from the ideal PCR sample (stored on ice from step 12) using a DNA clean and concentrator kit from Zymo Research, following the manufacturer’s protocol. This step removes PCR primers, which can interfere with quantitation and subsequent REPSA selection rounds.
17. Quantify the purified PCR products using a Qubit fluorometer (29). This purified dsDNA will now be used as the input DNA for the next round of REPSA.

### Subsequent rounds of REPSA.

The above steps from “Round 1 of REPSA” are repeated using the purified PCR products from the previous round. For example, the binding and control reactions for round 2 of REPSA will each contain 3 ng of the PCR products purified from either 6, 9, or 12 cycles of PCR from the round 1 +TF reaction. The purified PCR products from round 2 +TF reactions will then be used as inputs for the binding and control reactions for round 3. This should continue until a cleavage-resistant species is identified in the +TF reaction sample by gel electrophoresis. This is exemplified in Fig. 2B, in which an uncut DNA population is identified in the +TF reaction in round 4. Typically, our laboratory identifies cleavage-resistant populations within 3 to 6 rounds of REPSA, although this is largely based on the binding affinity of the transcription factor used and the complexity of the DNA-binding sequence. As mentioned previously, sequential rounds of REPSA should alternate IISRE enzymes to avoid selection of IISRE-specific cleavage-resistant DNAs. An overview of each round of REPSA used in our example is presented in Fig. 2C, and gel electrophoresis images from each round of REPSA are presented in Fig. S1.

### Validation of cleavage-resistant DNA population.

Once a cleavage-resistant DNA population is identified, PCR products from that round should be purified and analyzed by traditional DNA-protein binding assays. These include the electromobility shift assay (EMSA) and/or the restriction endonuclease protection assay (REPA) (32–34). Selection template libraries (used as inputs for round 1 of REPSA) should be used as a negative control for these assays to show that the tested transcription factor specifically associates with the DNAs produced by later rounds of REPSA. An example of T. thermophilus PaaR binding to round 4 DNAs using EMSA is presented in Fig. 3A. Once binding of the DNA population is further validated, significant sequence motifs can be identified by high-throughput sequencing technologies and motif-based sequence analysis. Facile generation of suitable fusion libraries is possible, given the modular nature of REPSA selection templates, and is dependent on the massively parallel sequencing platform being used (e.g., Ion Torrent semiconductor sequencing, Illumina sequencing by synthesis). An example of a consensus sequence logo identified from sequencing round 4 DNAs from the example used throughout this protocol is presented in Fig. 3B. As with *in vitro* binding assays, the original selection template library should also be sequenced as a negative control. A sample motif output from sequencing a random selection template is included in Fig. S2.
