# Supplementary material for: Using Restriction Endonuclease, Protection, Selection, and Amplification to Identify Preferred DNA-Binding Sequences of Microbial Transcription Factors
Source: Microbiol Spectr. 2023 Jan 5;11(1):e04397-22. doi: 10.1128/spectrum.04397-22 (PMC9927371; doi:10.1128/spectrum.04397-22)
Supplement: Supplemental file 1 — Supplemental material. Download spectrum.04397-22-s0001.pdf, PDF file, 0.5 MB [file spectrum.04397-22-s0001.pdf]

Supplemental Information for:

Using restriction endonuclease, protection, selection and amplification (REPSA) to identify preferred DNA-binding sequences of transcription factors

John K. Barrows and Michael W. Van Dyke#

Department of Chemistry and Biochemistry, Kennesaw State University, Kennesaw, Georgia, USA

#Address correspondence to Michael W. Van Dyke, mvandyk2@kennesaw.edu.

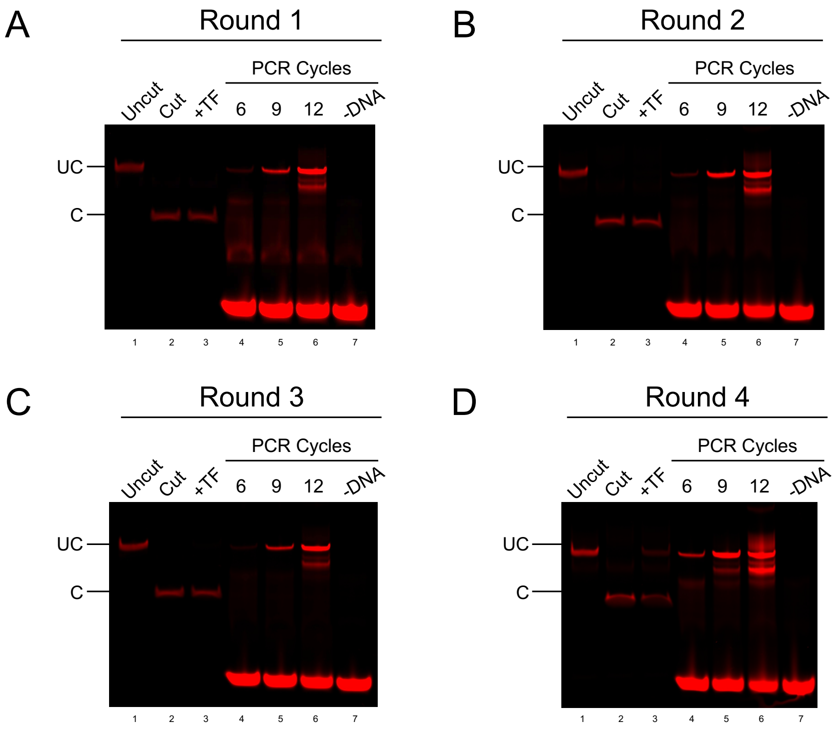

Figure S1: All rounds of REPSA with *Thermus thermophilus* PaaR. (**A – D**) Samples from the Binding/IISRE reactions and PCR reactions from each round of REPSA were separated by native PAGE and visualized using a LI-COR Odyssey Imager. Uncut (UC) and cut (C) DNAs from the IISRE reactions (lanes 1 – 3) are indicated. (Uncut) Reactions lacked PaaR and IISRE. (Cut) Reactions lacked PaaR and were challenged with 0.4 units IISRE. (+TF) Reactions contained 100 nM PaaR and were challenged with 0.4 units IISRE. DNAs from the +TF reaction were subject to the indicated cycles of PCR amplification (lanes 4 – 6). To identify any DNA contamination, PCR reactions lacking input DNA were amplified for 12 PCR cycles (-DNA; lane 7).

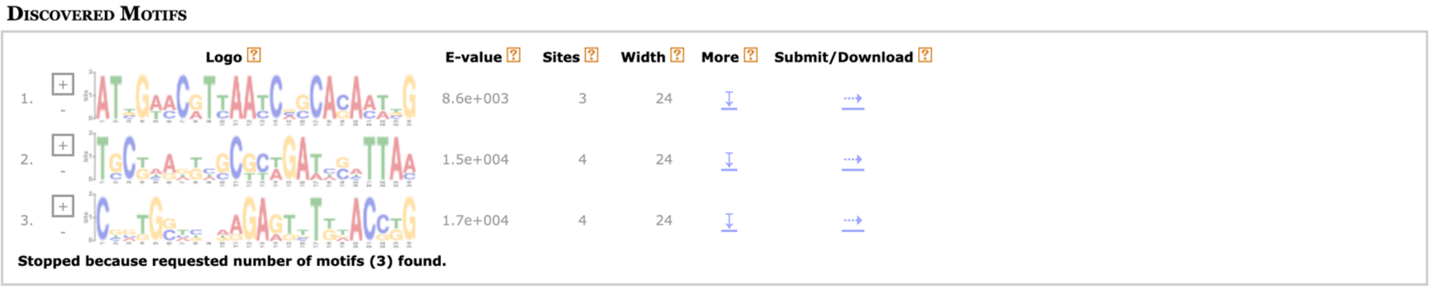

Figure S2: Selection template libraries yield no significant DNA motifs. PCR-amplified selection template DNAs were given sequence barcodes by fusion PCR to allow for massively parallel semiconductor sequencing using an Ion Torrent Personal Genome Machine. Resulting sequences were trimmed to yield only the 24-bp variable region. Approximately 8,000 sequences were then input into the web version 5.5.0 of Multiple Em for Motif Elicitation (MEME). A screen shot of the three most significant motifs is shown. Note that all E-values are much greater than the typical cut-off for significance, 0.05.
